# Clinical and molecular characterization of patients with adenylosuccinate lyase deficiency

**DOI:** 10.1186/s13023-021-01731-6

**Published:** 2021-03-01

**Authors:** Gerarda Mastrogiorgio, Marina Macchiaiolo, Paola Sabrina Buonuomo, Emanuele Bellacchio, Matteo Bordi, Davide Vecchio, Kari Payne Brown, Natalie Karen Watson, Benedetta Contardi, Francesco Cecconi, Marco Tartaglia, Andrea Bartuli

**Affiliations:** 1 Rare Diseases and Medical Genetics Unit, Academic Department of Pediatrics (DPUO), Bambino Gesù Children’s Hospital, IRCCS, Piazza Sant’Onofrio, 4, 00165 Rome, Italy; 2 Genetics and Rare Diseases Research Division, Bambino Gesù Children’s Hospital, IRCCS, Rome, Italy; 3 Department of Biology, University of Rome Tor Vergata, Rome, Italy; 4 Onco-Haematology and Cellular and Gene Therapy Research Division, Bambino Gesù Children’s Hospital, IRCCS, Rome, Italy; 5 Parents of patients affected by Adenylosuccinate lyase deficiency, Patient’s Association “Our Journey with ADSL deficiency”, Rome, Italy

**Keywords:** Adenylosuccinate lyase deficiency, Exome sequencing, Intellectual disability, Epilepsy, Neurometabolic disease, Purine nucleotide cycle defect

## Abstract

**Background:**

Adenylosuccinate lyase deficiency (ADSLD) is an ultrarare neurometabolic recessive disorder caused by loss-of-function mutations in the *ADSL* gene. The disease is characterized by wide clinical variability. Here we provide an updated clinical profiling of the disorder and discuss genotype–phenotype correlations.

**Results:**

Data were collected through "Our Journey with ADSL deficiency Association" by using a dedicated web survey filled-in by parents.

Clinical and molecular data were collected from 18 patients (12 males, median age 10.9 years ± 7.3), from 13 unrelated families. The age at onset ranged from birth to the first three years (median age 0.63 years ± 0.84 SD), and age at diagnosis varied from 2 months to 17 years, (median age 6.4 years ± 6.1 SD). The first sign was a psychomotor delay in 8/18 patients, epilepsy in 3/18, psychomotor delay and epilepsy in 3/18, and apneas, hypotonia, nystagmus in single cases. One patient (sibling of a previously diagnosed child) had a presymptomatic diagnosis. The diagnosis was made by exome sequencing in 7/18 patients. All patients were definitively diagnosed with ADSL deficiency based on pathogenic variants and/or biochemical assessment. One patient had a fatal neonatal form of ADSL deficiency, seven showed features fitting type I, and nine were characterized by a milder condition (type II), with two showing a very mild phenotype. Eighteen different variants were distributed along the entire *ADSL* coding sequence and were predicted to have a variable structural impact by impairing proper homotetramerization or catalytic activity of the enzyme. Six variants had not previously been reported. All but two variants were missense.

**Conclusions:**

The study adds more details on the spectrum of ADSLD patients’ phenotypes and molecular data.

## Background

Adenylosuccinate lyase deficiency (ADSLD) (MIM #103050) is a rare autosomal recessive disease first described by Jaeken and van den Berghe in 1984 [1], who found succinylpurines in the cerebrospinal fluid (CSF), plasma, and urine of three children with severe psychomotor delay and autism spectrum disorder (ASD). These succinylpurines, succinyladenosine (S-Ado) and succinylaminoimidazolecarboxamide riboside (SAICAr), are the dephosphorylated derivatives of ADSL substrates. The enzymatic defect is caused by mutations in the *ADSL* gene (MIM *608222), spanning over 23 kb on chromosome 22q13.1–13.2 [2, 3] 

Since the first *ADSL* mutation described [4], approximately 50 pathogenic/likely pathogenic variants and more than 100 clinically/functionally unclassified variants have been reported in the ClinVar (https://www.ncbi.nlm.nih.gov/clinvar) and ADSLD (http://www1.lf1.cuni.cz/udmp/adsl) databases, the majority being missense changes. Among the pathogenic variants, c.1277G > A (R426H) represents the most common event [5, 6]. Based on the available data, half of the patients with molecularly confirmed ADSLD are compound heterozygotes.

The ADSL enzyme is a homotetramer with a subunit size of ~ 50 kDa consisting of four active sites, each of which is contributed by three distinct subunits (PDB 2VD6) [7]. ADSL catalyzes two non-sequential steps in the purine biosynthesis: (1) the conversion of SAICA ribotide (SAICAR) into aminoimidazole carboxamide ribotide (AICAR) in the de novo purine synthesis, and (2) the conversion of succinyladenosine monophosphate (AMPS) to adenosine monophosphate (AMP) in the purine nucleotide cycle.

Although a wide clinical variability has been described, three different major phenotypes have emerged over the years. The fatal neonatal form is characterized by encephalopathy, intractable seizures, and respiratory failure, which lead to early death [8–10]. The severe form (type I) includes severe psychomotor delay, early onset of seizures and autistic features [10]. A moderate/mild form (type II) is characterized by later onset, mild/moderate psychomotor delay, and transient contact disturbances. Seizures, if present, appear later [11, 12]. Recently, a very mild phenotype characterized only by isolated psychomotor delay was described [13]. The pathogenic mechanisms are not yet fully understood, but it is likely to be related to the variable impact of mutations and the extent of residual enzymatic activity. However, the only biochemical marker that seems to correlate with the severity of the disease is S-Ado/SAICAr ratio in body fluids: the lower the ratio, the more severe the clinical symptoms of the patients are (fatal neonatal form S-Ado/SAICAr ratio in CSF < 1, type I ratio ~ 1, type II ≥ 2) [12, 14, 15].

The non-specific clinical spectrum and lack of awareness of the condition may prevent the correct and prompt diagnosis, which is essential for improving outcomes and quantifying reproductive risk.

This study aims to delineate clinical features of ADSL from a cohort of affected patients and investigate genotype–phenotype correlation.

## Results

### Clinical features

Over one year, 18 patients (12 males, age range six months—17 years, median age 10.9 years ± 7.3 SD, median 9.9 years), belonging to 13 unrelated families (including three pairs of siblings and a family with three affected siblings) from different geographical areas (two families from Italy, four from the USA, two from Poland, one each from France, Great Britain, Germany, Qatar and Israel) were enrolled. There was one case of consanguinity (patient 4) with first-degree cousin parents.

The age at onset was between birth and the first three years of life (median age 0.6 years ± 0.8 SD, median 0.33 years) but the age at diagnosis varied between two months and 17 years (median age 6.4 years ± 6.1 SD, median 3.5 years). The diagnostic delay was about six years (median age of 5.8 years ± 5.7 SD, median 3.3). In two cases having a very mild and non-specific phenotype, patients had to wait for 13 years to reach a diagnosis. The first noted sign/symptom was psychomotor delay in 8/18 patients (44.4%), epilepsy in 3/18 (16.7%), psychomotor delay and epilepsy in 3/18 (16.7%). One patient was asymptomatic at the time of enrollment and 3/18 patients (16.7%) had other symptoms (apneas, hypotonia, nystagmus).

Cardiotocographic abnormalities were detected in only one patient (patient 9). Some patients showed minor craniofacial features (thin upper lip, spaced teeth).

The diagnosis was made by whole-exome sequencing (WES) in 7/18 patients (38.9%), by biochemical analysis in 8/18 (44.4%), and by the combination of the two approaches in 2/18 (11.1%). In one patient, the diagnosis was attained by segregation analysis.

One patient presented fatal neonatal form, seven type I (38.9%), and nine type II (50%), two of which with a new very mild phenotype. The newborn with fatal form (patient #14) was a Qatar child, born at 37 weeks of gestational age from unrelated parents; he presented hypotonia, severe apneas, respiratory failure, microcephaly, drug-resistant epilepsy characterized by myoclonic seizures (more than 20 seizures/week), and severe psychomotor delay. He needed mechanical ventilation. He died at the age of 15 months. WES analysis documented the presence of two *ADSL* VoUS in compound heterozygosity, c.1343_1345delCTT (Ser448del), and c.502G > A (Val168Ile). Urine samples showed the presence of S-Ado (208.4, normal range 0–5 mmol/mole creatinine), SAICAr (223.3, normal range 0–0.8 mmol/mole creatinine) and S-Ado/SAICAr less than 1.

The seven patients with type I, including two siblings, had an early onset of disease (birth-nine months). In most cases, they showed an onset of epilepsy within the first six months of life. Severe cognitive and language delay and seizures were present in all patients. The epileptic phenotype included generalized tonic–clonic seizures, partial seizure, absence epilepsy, myoclonus and atonic seizure. The frequency of seizures was variable, ranging between 1 and 5 episodes/week and 11–15/week. Treatment, generally multidrug therapy, resulted in a complete response in one patient and partial response in four cases. Two patients had drug-refractory epilepsy. Neurologically, symptoms included hypotonia associated with spasticity in more than half of cases. The eye phenotype included strabismus and nystagmus. Cranial magnetic resonance imaging (MRI) showed cerebral atrophy in six patients. Microcephaly was reported in only one patient. Three patients had ASD, and behavioral abnormalities (hyperactivity, self and hetero-aggressiveness, easy irritability, stereotypies, inappropriate laughter) were reported in some patients, while poor eye contact was reported in all patients. Sleep apneas were observed in two patients, with the need for mechanical ventilation for one of the two. Three needed oxygen therapy in the post-critical period.

Table 1 summarizes the clinical, biochemical, and molecular data of patients with the severe form of ADSLD.

**Table 1:** Summary of clinical, biochemical and molecular data of seven patients with ADSL type I

Pt	Sex	Onset	Age of Diagnosis	First sign	PMR	Tone	Response to anticonvulsive treatment	ASD*	MRI	Eye phenotype	S-Ado CSF	Genotype	Head circumference (centile)
2	M	At birth	17 years	PMR	++	Hypotonia + spasticity	Partial	No	Cerebral and cerebellar atrophy	Strabismus	456.67	R426H/R426H	50
3	M	8 weeks	16 years	Seizure	+	Hypotonia + spasticity	Complete	No	Cerebral atrophy	Strabismus	856.43	R426H/R426H	50–85
4	M	4 months	7 months	PMR + seizure	+++	Hypotonia + spasticity	Partial	No	Cerebral atrophy	Strabismus	673.49	R426H/R426H	50–85
5	F	1 month	2 years	Seizure	+++	Hypotonia + spasticity	Refractory	No	Cerebral atrophy	No	ND	R149G/W175C	< 3
6	F	6 months	7 years	PMR	+	No	Partial	Yes	No	Strabismus	ND	Y114H/G418A	85
9	M	9 months	5 years	PMR	++	Hypotonia	Refractory	Yes	Cerebral atrophy	Strabismus + Nystagmus	ND	R141W/P318L	50
17	M	3 weeks	6 years	Seizure	+++	Hypotonia	Partial	Yes	Cerebral atrophy + hypomyelination	Strabismus + Nystagmus	311	S23R/R426H	3–15

ASD, autism spectrum disorder. CSF, cerebrospinal fluid. CSF values are in nanomoles/L. MRI, magnetic resonance imaging. ND, not done. PMR, psychomotor retardation: +, walking unassisted after 3 years, ++, never acquired standing position and unassisted walking, +++, never acquired sitting position

^*^All patients presented poor eye contact; patients 3, 6, and 9 showed stereotypies; patients 2, 3, 6, and 9 had inappropriate laughter; patients 3, and 6 showed self and hetero-aggressiveness

The nine patients with type II, including two pairs of siblings, presented mild to moderate neurodevelopmental and speech delay. Among them, two siblings from Germany showed a very mild isolated psychomotor delay without other manifestations; they also achieved numerous personal autonomies of daily life and showed an improvement in language and motor skills [13]. Six children of type II group developed seizure (generalized tonic–clonic seizures, clonic seizures, absence epilepsy). The epileptic crisis was sporadic or occurred with a frequency of 1–5 episodes/week. All patients showed an effective response to antiepileptic therapy; two children had a complete response. Neurologically, five patients presented hypotonia, associated with spasticity in one case. Eye disorders were also common in this group, mainly strabismus. Besides two children had nystagmus.

Brain MRI showed cerebral atrophy and hypomyelination in only one patient, as well as microcephaly. Four patients presented ASD and four children stereotypies. No respiratory problems were reported.

The apparently asymptomatic patient (#13), sibling of patient #12, was a child of 4 months at the time of enrollment. She was born at 42 weeks of gestational age from unrelated parents. The diagnosis was made at two months by *ADSL* gene sequencing, which documented the presence of the homozygous c.1277G > A missense change (p.Arg426His).

Table 2 summarizes the clinical, biochemical, and molecular data of patients with moderate/mild and very mild form of ADSLD.

**Table 2:** Summary of clinical, biochemical and molecular data of nine patients with ADSL type II

Pt	Sex	Onset	Age of diagnosis	First sign	PMR	Tone	Seizure		Response to anticonvulsive treatment		ASD*	MRI	Eye phenotype	S-Ado urine	Genotype	Head circumference (centile)
1	F	6 months	9 years	PMR + seizure	+	Hypotonia	Yes	Good		No		Normal	Strabismus + Nystagmus	115.45	R309H/c.1191 + 5G > C	50–85
7	M	6 months	4 years	PMR	+	Normal	Yes	Good		Yes		Normal	Strabismus	ND	Y114H/G418A	85–97
8	M	6 months	2 years	PMR	+	Normal	Yes	Good		Yes		Normal	Normal	322.4	Y114H/G418A	85–97
10°	M	2 years	17 years	PMR	+	Hypotonia	No	NA		No		Normal	Normal	22.3	M26L/R396H	97
11°	F	2 years	13 years	PMR	+	Hypotonia	No	NA		No		Normal	Normal	19.9	M26L/R396H	97
12	M	At birth	2 years	Nystagmus + PMR + congenital torticollis	++	Hypotonia + spasticity	No	NA		Yes		Cerebral atrophy + hypomyelination	Strabismus + Nystagmus	ND	R426H/R426H	50
15	M	At birth	3 years	Hypotonia	+	Normal	Yes	Good		No		Normal	Normal	122.5	E343K/E343K	97
16	M	4 months	3 years	PMR	+	Hypotonia	Yes	Good		Yes		Normal	Strabismus	469	R426H/D430D	3
18	F	3 years	12 years	PMR + seizure	+	Normal	Yes	Complete		No		Normal	Normal	175.95	T450S/D332H	97

ASD, autism spectrum disorder. MRI, magnetic resonance imaging

*Four patients presented sterotypies, Patient 12 with poor eye contact, aggressiveness and hyperactivity. Patients 12 and 16 with inappropriate laughter. PMR, psychomotor retardation: +, walking unassisted after 3 years, ++, never acquired standing position and walking unassisted, +++, never acquired sitting position. Urinary values are in millimoles/mol creatinine. °Patients with a new very mild phenotype

### Biochemical data

Urine samples of nine patients showed high levels of SAICAr and S-Ado. Analysis of cerebrospinal fluid was performed in four patients and evaluation of enzyme catalytic activity in erythrocytes in two patients (Table 3).

**Table 3:** Biochemical data of patients with ADSL

Patient	S-Ado urine	SAICAr urine	S-Ado/SAICAr urine	S-Ado CSF	SAICAr CSF	S-Ado/SAICAr CSF	Enzyme catalytic activity	Phenotype
1	115.5	57.6	2	–	–	–	81.95 (− 25%)	Moderate/Mild
2	–	–	–	456.7	–	–	–	Severe
3	–	–	–	856.4	–	–	–	Severe
4	–	–	–	673.5	–	–	–	Severe
5	Positive°	–	–	–	–	–	–	Severe
8	322.4	240.6	1.3	–	–	–	–	Moderate/Mild
9	–	–	1.4	–	–	–	–	Severe
10	22.3	6.2	3.6	–	–	–	–	Very mild
11	19.9	5.4	3.7	–	–	–	–	Very Mild
14	208.4	223.3	< 1	–	–	–	–	Fatal neonatal
15	122.5	–	–	–	–	–	–	Moderate/Mild
16	469	–	–	–	–	–	–	Moderate/Mild
17	–	–	–	311	374	0.8	–	Severe
18	176	72.8	2.4	–	–	–	74.1 (− 20%)	Moderate/Mild

CSF, cerebrospinal fluid. CSF values are in nanomoles/L

Urinary values are in millimoles/mol creatinine. Enzyme catalytic activity is in UI/ erythrocytes

°, value not indicated

### Molecular data

Based on the American College of Medical Genetics and Genomics criteria [16], one pathogenic variant (class 5), seven likely pathogenic (class 4) and ten VoUS, including six novel variants, were reported (Additional file 1). Segregation study was performed in all cases confirming the homozygosity/compound heterozygosity status and the heterozygous status of each parent. The vast majority of variants were missense (34/36 variants); an in-frame deletion (c.1343_1345delCTT, p.Ser448del) and one splice site variant (c.1191 + 5G > C) [17] were reported in single patients.

The recurrent R426H substitution was identified in seven patients (38.8%), either as homozygous change (five cases) or in compound heterozygosity with a second variant in two children. The six novel variants affected different regions and functional domains of the protein. Positive biochemical test in children carrying novel variants (patients 1, 5, 14, 15, 18) confirmed their causative role in disease presentation and their bona fide pathogenic impact.

### Structural impact of the previously unreported ADSL pathogenic variants

**Fig. 1:**
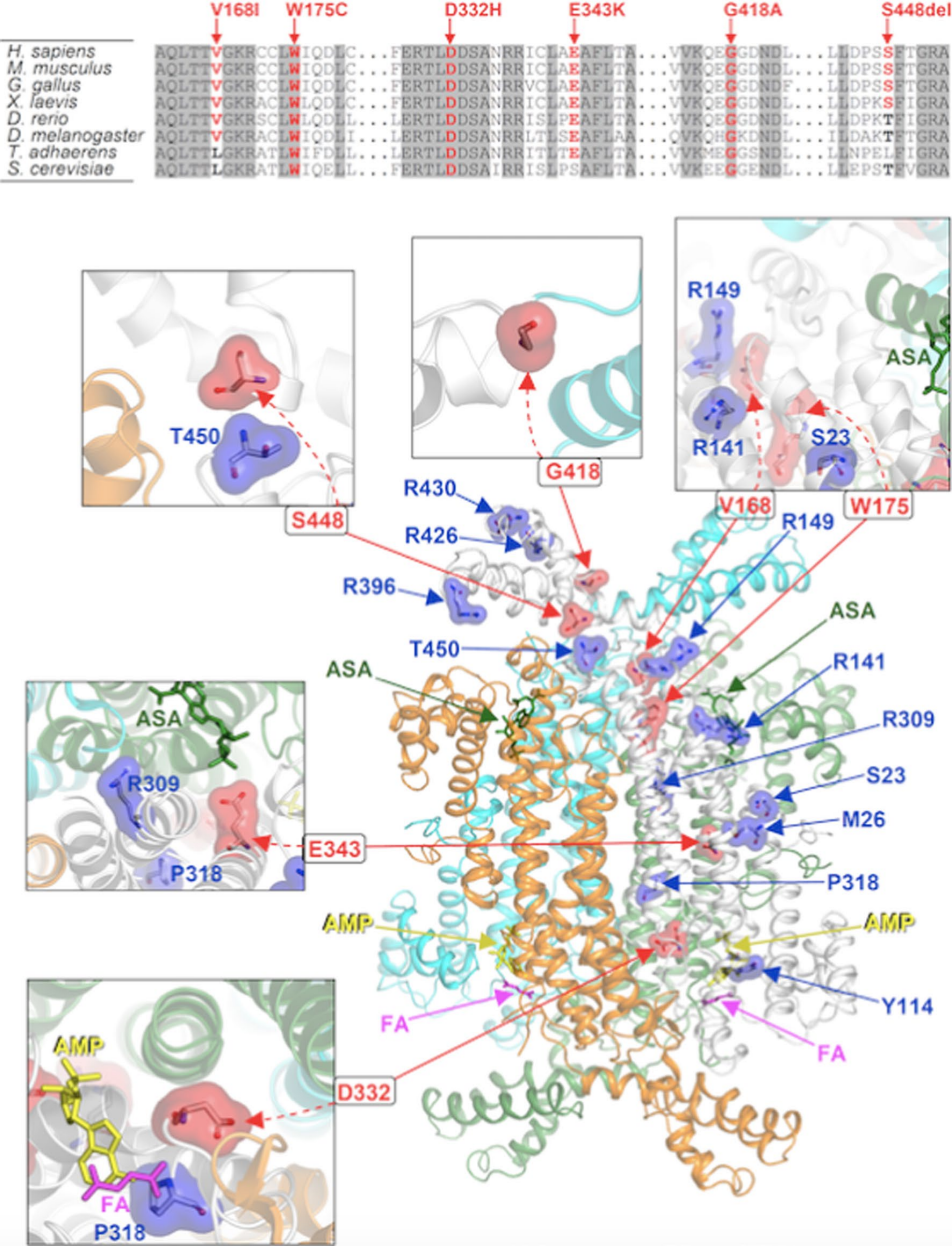
Sequence alignments and location of the mutated residues in the identified ADSL holoenzyme organization. (top) Multiple sequence alignments around the residues affected by the previously unreported *ADSL* mutations (columns with invariant residues are grayed). (bottom) Crystal structure of the ADSL homotetramer (PDB 2VD6) showing the amino acids hit by mutations (the residues affected by the novel mutations presented in this study are highlighted by a red surface, those affected by previously reported a blue surface highlights mutations; all mutation sites are mapped on the same monomer; the four monomers are in different ribbon colors). The co-crystallized substrate and its enzymatic products (adenylosuccinic acid, ASA, green sticks; adenosine monophosphate, AMP, yellow sticks; fumaric acid, FA, magenta sticks) are also shown. The closed-up views highlight structural details around the sites involved by the novel mutations (including the known mutations falling nearby). For better clarity of visualization of the residues, enlarged views are oriented differently from the whole structure. Amino acid numbering of ADSL protein refers to the NCBI protein entry NP_000017.1

The location of the newly reported variants in the structural model of homotetrameric ADSL is illustrated in Fig. 1.

The six novel variants were predicted to have a similar structural/functional impact on the ADSL holoenzyme by affecting the proper oligomerization of the ADSL monomer. V168I is invariant from humans to insects and replaced by a similar residue (leucine) in lower organisms. Val168 interacts with Arg149, which is the site of the previously reported disease-causing Arg149Gly substitution. Nevertheless, it was predicted to cause conformational changes at the nearby region of interaction with another ADSL monomer. Similarly, the highly conserved Trp175 is also close to the region of interaction with another ADSL monomer; W175C change is non-conservative and is expected to induce significant conformational changes impairing the homotetramerization. D332H substitution should be detrimental to ADSL functions since it represents a non-conservative change involving the invariant Asp332 at the region of tetramerization and in the proximity of the binding region of the enzymatic products adenosine monophosphate and fumaric acid. The E343K change implies electrostatic charge inversion inside the region of tetramerization and is predicted to cause defects in this process. Similarly, the invariant Gly418 residue contributes to the stabilization of the ADSL tetramer. The methyl side chain introduced by the G418A mutation decreases the flexibility of the affected protein site in an ADSL monomer and also introduces novel interactions with residues of another bound ADSL monomer; thus, it could have an essential effect on tetramerization.

Finally, Ser448 lies near the region of interaction with another ADSL monomer. Although Ser448 is conserved only in higher organisms (and replaced by a similar residue, threonine, or leucine in phylogenetically distant species), the effect of its deletion is to perturb the ADSL structure locally and impair ADSL tetramerization considerably.

## Discussion

In this study, we describe parent-reported clinical, biochemical, and molecular features of 18 patients affected by ADSLD.

The majority of patients described so far presented a severe clinical phenotype, with only about 15–20% of cases with a milder form [18]. While our study is limited by recruitment among families participating in a patient organization, the present cohort documents a considerably higher frequency of type II ADSLD (50%), which suggests that this form is more common than previously appreciated. Patients with type II of ADSLD may not present an obvious sign of disease progression, and degradation and symptoms may be milder and non-specific. Due to a lack of specific features, the diagnosis of attenuated patients is difficult, and many cases probably remain undiagnosed.

Despite an early onset of the disease, diagnosis is often delayed, leading to a "diagnostic odyssey" for young patients and their families. Indeed, albeit an early manifestation of the isolated psychomotor delay was already observed before the age of two in more than half of the cohort, the average age at diagnosis was much later, at about six. DNA sequencing techniques have partially solved this problem. In recent years, the development of modern technologies for sequencing the human genome, together with the growth of knowledge on genetics, has made available genetic tests for molecular diagnosis of Mendelian conditions characterized by marked clinical heterogeneity and partial overlapping of phenotypes [19, 20]. Therefore, WES has become part of routine clinical practice.

In our cohort, 7 out of 18 patients were diagnosed by WES, and by the two combined methods (WES and biochemical test) in two other patients, almost all had a type II form, suggesting that more patients with mild/nonspecific presentations could be identified "genotype first" approaches that are less affected by selection bias.

The collected data indicate that type II cases have a mild neurological involvement with absence or a lower incidence of epilepsy, in line with results reported by Jurecka et al. [18]

In this cohort, the frequency of ASD is similar in the two phenotypic classes, while there is a higher percentage of brain imaging anomalies in the severe group. In literature, various anomalies in brain tissue, including cerebral cortex atrophy, corpus callosum agenesis, delayed or absent myelination, white matter anomalies and lissencephaly, were reported in all three phenotypic classes [21, 22]. In a previous series of seven patients [21], all patients with type I had brain anomalies and microcephaly since the first year of life; type II patients had milder, non-specific brain anomalies without microcephaly. According to these findings, the authors suggested that microcephaly may be secondary to brain alterations. Our data do not support this hypothesis. While the prevalence of alterations in terms of frequency and severity in type I phenotype was confirmed, microcephaly was detected in only one patient with a severe phenotype.

ASD was initially reported as a typical sign of ADSLD and found in about a third of patients [1, 23]. In our cohort, 40% had an ASD diagnosis. Behavioral abnormalities, including hyperactivity, self and hetero-aggressiveness, inappropriate laughter were frequently reported, particularly in type I. In type II group, epilepsy had an earlier onset and responded better to therapy. Frequency of muscle tone and ocular abnormalities were consistent with previous data [6]; hypotonia was often associated with spasticity, especially in patients with severe form.

Similar to previous reports [24], craniofacial dysmorphisms were absent or mild and non-specific.

Therefore, our data confirmed the wide clinical heterogeneity. As previously reported, two German siblings showed a very mild isolated psychomotor retardation without other manifestations defining a new phenotype [13].

More than 150 variants of *ADSL* gene have been described, and most of them are missense changes (ClinVar database). In our cohort, we identified 34 missense variants, confirming an evident prevalence of these variants compared to other types. Nonsense variants represent only a small fraction (< 5%) of all the pathogenic *ADSL* variants reported, probably because complete loss of ADSL function is incompatible with development. The most common disease-associated variant, c.1277G > A (R426H), leading to structural instability of the enzyme [25], has been identified in about 40% of patients. This recurrent nucleotide substitution was observed to occur at the homozygous state in five cases (three of whom showing a severe phenotype) and at the heterozygous state in two patients; when it was associated with another known class 5 mutation, the phenotype was moderate/mild, while when associated with a novel variant, the phenotype was severe. R396H and Y114H mutations, described to lead to severe functional effects, were unexpectedly associated with a milder phenotype. Similar to previous reports, no clear genotype–phenotype correlation about these mutations has been observed.

Only slightly more than 50 of all *ADSL* variants reported are classified as pathogenic according to the ACMG criteria, while more than 50% are classified as VoUS. Ten VoUS (10/36) have been identified in the cohort; eight of them were in compound heterozygosity defining a not previously reported genotype. In all subjects carrying these variants, a biochemical assessment confirmed ADSLD diagnosis, validating the clinical relevance of the identified variant.

Structural inspection of the disease-associated variants documented that their impact on proper oligomerization of the ADSL monomer is a general feature. Structural considerations, however, do not allow to appreciate the specific impact of a subset of identified variants. This is exemplified by the predicted structural consequences of the E343K substitution, which was identified as a homozygous change in a patient with a mild phenotype. The side chain of Glu343 of each ADSL monomer is in contact with the side chain of Glu343 located in another monomer, producing potential electrostatic intermolecular repulsions. Although the E343K mutation implies an anionic to cationic electrostatic charge inversion of the affected site, homozygosity, by inverting all Glu343 anions to Lys343 cations, results in the "conservation" of repulsive intermolecular interactions among ADSL monomers. Since Glu343 side chains are located at the monomer–monomer interfaces but do not present further interactions with other residues, we are not able to predict significant detrimental effects for the E343K mutation. The "conservation" of repulsive-type forces in ADSL dimers and tetramers might explain the mild phenotype associated with E343K in homozygosis.

The two novel variants, Ser448del and V168I, were associated with a fatal neonatal form of ADSLD. In particular, Ser448del is detrimental to ADSL tetramerization; it could, therefore, determine more severe functional consequences and explain the phenotype severity.

The variant W175C is expected to destroy the protein fold for its location and type of amino acid replacement, as well as the R149G mutation, has an important structural role. This might explains the severe phenotype presented by the patient carrying the two mutations on distinct alleles.

Intriguingly, the novel variant D332H should be detrimental to ADSL function; however, in one patient, D332H is in compound heterozygosity with T450S mutation, and it is associated with a mild phenotype. This allows hypothesizing the presence of factors that may mitigate the final phenotype or, most likely, the T450S mutation may preserve a residual enzymatic activity.

There is no effective treatment for ADSLD; only a few reports propose therapeutic strategies to prevent intractable epilepsy. Of these, two showed some beneficial effects [26, 27], not confirmed by subsequent studies [28, 29].

## Conclusions

In conclusion, our study adds more details on the spectrum of ADSLD phenotypes. Our experience also underlines the crucial role of patient/parent association as a critical resource to connect families who share common experiences, collect patient information, and support pediatricians in diagnostic tools. Furthermore, genotype characterization may help to define the biological effects of novel variants better. A considerable effort is still needed to understand the molecular mechanism on cellular metabolism, thus fostering the development of therapeutic strategies to limit the adverse effects on the nervous system and muscles.

## Methods

Patients were enrolled through the Patient's Association "Our Journey with ADSL deficiency." Families were advised through the association's Facebook Page of the possibility of participating in the study. Parents who expressed their interest were contacted by clinicians, who collected the informed consent and administered the information sheet. Data collection was carried out through a survey filled directly by patients' parents. The survey, built on a literature review, is aimed to investigate clinical, biochemical, and molecular features of affected patients (Additional file 2).

The data relating to each patient were processed anonymously in order to guarantee privacy according to current legislation. Data were collected into an in-house database and managed exclusively by researchers involved in the study project.

Three patients have been previously reported in the literature [13, 17].

The study was conducted following the principles of the Declaration of Helsinki and received the approval of the local Ethics Committee (DALIstudy—1973_OPBG_2019).

### Structural analysis

Our genotype–phenotype correlation is based on a structural analysis that consisted of the visual inspection of the amino acids affected by mutations within the crystal structure of the ADSL protein, tetramerized and complexed with its substrate and catalytic products (Protein Data Bank, PDB, code 2VD6). This allowed us to observe that the various mutations directly affect (and alter) functional regions of ADSL (either catalytic sites, or ligand binding residues, or protein regions involved in tetramerization) and consequently we envisaged the effects as explained in detail in the text.æ

Molecular graphs were made with PyMOL (www.pymol.org).

## Supplementary Information

**Additional file 1.** ADSL variants in silico evaluation.

**Additional file 2.** ADSL survey aimed to investigate clinical, biochemical, and molecular features of affected patients.

## Data Availability

Data sharing is not applicable to this article as no datasets were generated or analysed during the current study.

## Abbreviations

ADSL: Adenylosuccinate lyase deficiency
AICAr: Aminoimidazole carboxamide ribotide
SAICAR: Succinyl-aminoimidazole carboxamide ribotide
AMP: Adenosine monophosphate
S-Ado: Succinyladenosine
S-AMP: Adenylosuccinate
MRI: Magnetic resonance imaging
WES: Whole exome sequencing
