# Supplementary material for: Clinical and molecular characterization of patients with adenylosuccinate lyase deficiency
Source: Orphanet J Rare Dis. 2021 Mar 1;16:112. doi: 10.1186/s13023-021-01731-6 (PMC7919308; doi:10.1186/s13023-021-01731-6)
Supplement: Supplementary file 1 — Additional file 1. ADSL variants in silico evaluation. [file 13023_2021_1731_MOESM1_ESM.doc]

**Parents contact:**

- **Mother**

surname

name

phone

email address

- **Father**

surname

name

phone

email address

**Affected Child**

**Surname:**

**Name:**

**Sex:**

Male

Female

**Date of birth:**

**Place of birth:**

**Age of onset:**

**First sign (please give details of signs and symptoms):**

**…..................................................................................................................**

**Age of diagnosis:**

**Who made the diagnosis?**

**In which Hospital?**

**Method used for obtain diagnosis ?**

**Family history (consanguinity/other relatives affected):**

**Do you have other children?**

Yes

**No**

**Have any other children in your family (immediate or otherwise) been diagnosed with ADSL deficiency?**

Yes

No

- If Yes:

Immediate

Other

**Is your child still alive:**

Yes

No If No, at wich age it pass away?..............

**Abnormal cardiotocography (CTG):**

Yes

No

**Neurologic features**

- Psychomotor delay (mild, moderate, severe):

1. Sitting up unassisted?

Yes

No

Almost / Understands the objective however is still practising

1. At what age did your child sit up unassisted?

Under 18 months old

Under 36 months old

Older than 36 months

1. Standing unassisted:

Yes

No

Almost / Understands the objective however is still practising

1. Walking unassisted

Yes

No

Almost / Understands the objective however is still practising

1. What age did your child stand or walk unassisted?

Under 36 months

Over 4 years old

Other (provide details)

- speech and language delay

Yes

No

- Mental retardation

Yes

No

- Hypotonia

Yes

No

- Poor eye contact

Yes

No

- Seizures:

Yes

No

1. At what age did they start to have seizures?

From new born

Under 3 months old

Within the first 6-12 months

Under 3 years old

Over 3 years old

Other, give details

1. Do you know of anything that may trigger the seizures? (Please give details, i.e. Diet-High Carbs, lack of sleep, changes in light)

….........................................................................................................................................

1. Seizure frequency (weekly basis):

1-5

6-10

11-15

More than 20 a week

Other

1. Does your child *currently* take medication for seizure management?

Yes

No

1. What medications does your child *currently* take to manage seizures? (Please also give dosage and frequency**)**
2. Seizure frequency with *current* drug therapy (weekly basis):

1-5

6-10

11-15

More than 20 a week

Other

1. Have these drugs improved your child's seizures management?

YES they are much less frequent

NO there is currently no reduction to seizures

YES they have stopped them completely

Somewhat but not completely

None of these options

1. What medications has your child taken in the past to manage seizures? (Please also give dosage and frequency**)**

-medication name:.......................... dosage:.................................time for day...................

from(date)............................. to(date)..........................

associated with other medication(name of the medication):...............................

effect on seizures....................................... side effects......................................................

-medication name:.......................... dosage:.................................time for day...................

from(date)............................. to(date)..........................

associated with other medication(name of the medication):...............................

effect on seizures....................................... side effects......................................................

-medication name:.......................... dosage:.................................time for day...................

from(date)............................. to(date)..........................

associated with other medication(name of the medication):...............................

effect on seizures....................................... side effects......................................................

1. What type of seizures does your child typically/frequently have?

"Grand Mal" or Generalized tonic-clonic unconsciousness, convulsions,

muscle rigidity

Absence Brief loss of consciousness

Myoclonic Sporadic (isolated), jerking movements

Clonic Repetitive, jerking movements

Tonic Muscle stiffness, rigidity

Atonic Loss of muscle tone

None of these options (please give details)

…...................................................................................................................................

1. Has your child had an EEG?

Yes

No

- Spasticity

Yes

No

- Opisthotonus

Yes

No

- Cerebral atrophy

Yes

No

- Cerebellar atrophy

Yes

No

- Hypomyelination

Yes

No

- Atrophy of corpus callosum

Yes

No

- Strabismus

Yes

No

- Nystagmus

Yes

No

**Behavioral Psychiatric Manifestations:**

- Autistic features

Yes

No

- Hyperactivity

Yes

No

- Aggressive behavior

Yes

No

- Temper tantrums

Yes

No

- Stereotypic movements

Yes

No

- Self-mutilation

Yes

No

- Happy demeanor

Yes

No

- Inappropriate laughter

Yes

No

**About Birth**

**- Gestational age (weeks and days):**

**- Weight at birth:**

**- Length at birth:**

**- Head circumference at birth:**

**- APGAR score (at 1 and 5 minute):**

**- Growth retardation:**

**Yes**

**No**

If YES Age of onset............

- **Stature (last measurement): centimeters .......................**

**Weight (last measurement): kilograms ........................**

**Head circumference (last measurement): centimeters ....................**

**Head and face:**

- Brachycephaly

Yes

No

- Microcephaly

Yes

No

- Prominent metopic suture

Yes

No

- Thin upper lip

Yes

No

- Long smooth philtrum

Yes

No

- Low set ears

Yes

No

**Respiratory problems:**

- Pulmonary hypoplasia:

Yes

No

- Mechanical ventilation

Yes

No

- Does your child have symptoms of sleep apnea?

Yes

No

- Has your child had a sleep study completed?

Yes (please give details)

No

- Do they require oxygen treatment?

Yes

No

- How often do they require oxygen treatment?

Hourly

Daily

At night while they sleep

Only after seizure episodes and in hospital

Other

- Does your child require Chest Physiotherapy?

Yes

No

**Other symptoms:**

**….................................................................................................**

**CT/RMN imaging:**

Yes (please give details)

No

**Have you tried Ketagenic or a modified form of Atkins/ketagenic diet for your child?**

Yes

No

How long?....................................

Was it helpful?

Yes

No

Somewhat

- In what way was it helpful or unhelpful?

…........................................................................................................................................

**Has your child currently been diagnosed with any other conditions?**

Yes (please give details)

No

**Important documents to complete the research**

***Please attach photos of the patient***

***Please attach the biochemical test reports below***

1. **S-Ado Plasma:**
2. **SAICAr Plasma:**
3. **S-Ado Urine (millimoles/mol creatinine):**
4. **SAICAr Urine (millimoles/mol creatinine):**
5. **S-Ado/SAICAr ratio:**

- Plasma
- Urine
- cerebrospinal fluid (CSF)

***Please attach the genetic test below and other exams required:***

1. **cDNA mutations:**
2. **Aminoacid substitutions:**
3. **Genetic test of parents and/or siblings:**
4. **Inheritance (compound heterozygous/homozygous):**
5. ***CT/RMN imaging***
