# Supplementary material for: Clinical and molecular characterization of patients with adenylosuccinate lyase deficiency
Source: Orphanet J Rare Dis. 2021 Mar 1;16:112. doi: 10.1186/s13023-021-01731-6 (PMC7919308; doi:10.1186/s13023-021-01731-6)
Supplement: Supplementary file 2 — Additional file 2. ADSL survey aimed to investigate clinical, biochemical, and molecular features of affected patients. [file 13023_2021_1731_MOESM2_ESM.docx]

| **With REVEL > 0.5 and CADD > 15 are pathogenic (M-CAP >0.025)** | | | | |  |  |
| --- | --- | --- | --- | --- | --- | --- |
| **Genomic hg19** | **nt change** | **aa change** | **m-CAP v.1.0** | **REVEL** | **CADD v.1.3** | **ACMG** |
| chr22-40757555-G-A | c.926G > A | Arg309His | 0.167180364 | 0.839 | 35 | Uncertain Significance |
| chr22-40760969-G-A | c.1277G>A | Arg426His | 0.221777465 | 0.901 | 27.5 | Pathogenic |
| chr22-40750294-C-G | c.445C>G | Arg149Gly | 0.209583605 | 0.553 | 24.2 | Uncertain Significance |
| chr22-40746022-T-C | c.340T>C | Tyr114His | 0.607583747 | 0.97 | 28.4 | Likely Pathogenic |
| chr22-40750270-C-T | c.421C>T | Arg141Trp | 0.690420949 | 0.904 | 34 | Likely Pathogenic |
| chr22-40742638-A-T | c.76A>T | Met26Leu | 0.412674543 | 0.872 | 24 | Likely Pathogenic |
| chr22-40761029-CTT- | c.1343_1345delCTT | Ser448del | NA | NA | 18.85 | Uncertain Significance |
| chr22-40759001-G-A | c.1027G>A | Glu343Lys | 0.283637121 | 0.832 | 29.2 | Uncertain Significance |
| chr22-40742631-C-G | c.69C>G | Ser23Arg | 0.575471104 | 0.882 | 32 | Uncertain Significance |
| chr22-40761041-C-G | c.1349C>G | Thr450Ser | 0.245673102 | 0.816 | 24.3 | Likely Pathogenic |
| chr22-40760374-G-C | c.1191 + 5G > C | - | NA | NA | 15.85 | Uncertain Significance |
| chr22-40754910-G-T | c.525G>T | Trp175Cys | 0.640784778 | 0.931 | 33 | Uncertain Significance |
| chr22-40760945-G-C | c.1253G>C | Gly418Ala | 0.115010865 | 0.882 | 27.8 | Uncertain Significance |
| chr22-40757582-C-T | c.953C>T | Pro318Leu | 0.086414024 | 0.679 | 32 | Uncertain Significance |
| chr22-40760365-G-A | c.1187G>A | Arg396His | 0.561257346 | 0.962 | 35 | Likely Pathogenic |
| chr22-40754887-G-A | c.502G>A | Val168Ile | 0.575331488 | 0.75 | 29.1 | Uncertain Significance |
| chr22-40760980-G-A | c.1288G>A | Asp430Asn | 0.108309866 | 0.659 | 32 | Likely Pathogenic |
| chr22-40757623-G-C | c.994G>C | Asp332His | 0.204237252 | 0.854 | 32 | Likely Pathogenic |
